# The Prevention of the Increase of Insanity, of the Procreation of the Congenitally Defective and of the Criminally Disposed

**Published:** 1912-10

**Authors:** Daniel Parker

**Affiliations:** Calvert, Texas


### TEXAS MEDICAL JOURNAL.

Established July, 1885

F. E. DANIEL, M. D., - - - - - Editor, Publisher and Proprietor

Published Monthly.—Subscription, $1.00 a Year.

VOL. XXVIII. AUSTIN, OCTOBER, 1912. No. 4.

The publisher is not responsible for the views of the contributors.

### Original Articles.

For Texas Medical Journal.

### The Prevention of the Increase of Insanity, of the Procreation of the Congenitally Defective and of the Criminally Disposed.

BY DANIEL PARKER, M. D., CALVERT, TEXAS.

*Gentlemen of the Texas State Medical Association:*

I desire to call your attention to a subject of the utmost importance to every citizen of the State of Texas, and that is the rapid increase of the number of the insane, and also that of the propagation of the congenital defective, as well as those with hereditary criminal tendencies.

The rapid increase in the number of the insane is alarming and largely out of proportion to the increase in our population. Our hospitals for the insane now cost the State close to the million-dollar mark annually, with the certainty of a steady increase unless present conditions can be changed. This lamentable state of affairs calls for the serious and earnest attention of every thinking man, and unless the attention of our Legislature can be effectually called to it, and means adopted to prevent in some measure the propagation of this class of defectives, it seems inevitable that the State will eventually be overwhelmed. This is a brief and I trust sufficiently comprehensive statement of the condition to which I wish to call your attention.

Let us first mention the law of heredity, which we might prop-

*This paper was intended for the Section on State Medicine, to be read at the Waco meeting of the Association in May, but was not received in time.

erly style "the first law of nature," since it was promulgated in the garden of Eden by the Great Creator, who decreed "That the earth bring forth the living creature after his kind." Whether this was really a divine decree, or the recording of a fact already established at that remote period, matters not; universal experience establishes the fact that "like produces like."

It is sufficient for the purposes of this paper to consider this law only as it affects the transmission of disease, and of abnormal mental and physical characteristics. The problem of caring for defectives and balancing the forces of environment and education so as to overcome a hereditary tendency does not come within the scope of this paper. We seek to prevent the propagation of this class, to eliminate them, so far as it is possible, from the human mass, to prevent them from being born. In the more primitive conditions of society the law of "the survival of the fittest" virtually accomplished this object. The weaklings, the degenerate not being able to withstand the rigors to which they were subjected, did not live to become a charge to the State, or to propagate their kind. In our higher state of civilization the pendulum has swung in the opposite direction. Our improved hygienic conditions and better methods of treatment have taken wealklings that should never have been born, and rescued them, to become a charge to the State, and, still worse, to add the burden of their defective progeny. This in my opinion largely accounts for the disproportionate increase of insanity.

I have no patience with the idea that it is good policy or good morals to permit, so far as it is possible to prevent it, the burdening of society with such as can only become a charge upon the public, and of unhappiness to themselves. If unfortunately such are born they are entitled to symathy and consideration, but they never should have been born, and, being born, they should not be allowed to lay additional burdens on society, by the procreation of their kind.

Population is only desirable when it is of the right kind. While a healthy normal child is an asset to society, a degenerate is a liability.

In regard to insanity, I believe that it is estimated that three-fourths of the insane have a hereditary taint which has finally brought them to some hospital for the insane for treatment. What a relief it would be to society and the State if the propagation of this class could be prevented. It is not contended that insanity can be prevented, but it is contended, and susceptible of proof, that the sterilization of this class would immensely relieve this unfortunate condition.

Criminal tendency is a species of insanity. Hardly anyone will contend that a well-balanced and normal mind would incite to commit crime. The physicians' records at the New York State Reformatory show that two-fifths of its inmates are mentally defective. This tendency, of course, may be more or less marked according to what might be called the degree of mental deformity, but the relief would be incalculable, if the propagation of this class could be prevented. The famous case of the Jukes family, where "Margurett the mother of criminals," a half-insane prostitute, had criminal descendants numbering several hundred, whose careers of crime included murder, thieving and prostitution. Further than that, no doubt, a criminal tendency developed in many of her descendants who themselves were considered respectable. If the ancestry of many of our criminals could be traced, unquestionably a vicious hereditary tendency could be found, perhaps cropping out after many generations.

The rational treatment of the congenital defective is equally obvious. Our State schools for the blind and the deaf are largely composed of descendants of defectives. These people receive their education, go home to marry, and in due time send their unfortunate progeny back to the State institutions, where they receive their education. If the congenitally defective could be eliminated from our population, those eleemosynary institutions would require very little assistance from the State, and society would be relieved of considerable burden.

All of these conclusions are sufficiently obvious. It seems almost Utopian after submitting to this unfortunate condition from time immemorial, as unavoidable, to hope that the hydra-headed evil of insanity, crime and congenital deformity can be successfully coped with; yet, in a properly organized state of society, it would be the simplest of all our social problems. These people should not be allowed to propagate their kind. No more of them should be born.

Sterilization by means of vasectomy is the remedy. That it is a remedy is not to be questioned, and all objection to it is purely sentimental. It deprives the subject of no power, except that of procreation, which is the object we seek to accomplish.

So far, I have considered this subject only from the standpoint of public policy, but the advantage to the subject is equally obvious. No doubt many an intelligent person who recognizes some hereditary defective tendency hesitates to enter into the marriage state, fearing defective progeny. If such a person could be assured that there would be no progeny, this relation could be entered into safely, and two persons could live together in conjugal happiness, with all the pleasures thereunto appertaining. Surely this is no mean consideration.

Another aspect of this operation, as it affects the criminal subject, is that it improves his general health, and renders the criminal tendency less dominant.

Dr. Woods Hutchinson states, in an article in *Everybody's Magazine,* that out of one hundred and six men set at liberty on parole, after being subjected to vasectomy, only five relapsed. This is a fine showing. Among the violently insane, this operation has had the effect of quieting the subject and rendering him much more manageable.

It is often said that there are two sides to every question. To me this seems an exception. So far as I can see it, it is all for and nothing against. I hardly need describe this operation to this body. It is simple, without danger, practically painless and absolutely certain to accomplish the object for which it is designed.

The outline of a law on this subject should be, in my opinion, something like this: All paroled and discharged patients from our State hospitals for the insane, with perhaps a few exceptions, should be sterilized before leaving the hospital. Certain classes of criminals should be submitted to the operation before being discharged or paroled, and no marriage license should be issued where either party has a congenital defective taint, unless one of the parties is sterilized. This is merely an outline.

Several States have already enacted laws on this subject. Indiana took the lead in 1907. Connecticut followed in 1909, and the province of Ontario in 1910. Other States have also legislated on this subject. The question now is: Shall Texas join the progressive column?

I introduced a bill in the last Legislature legalizing this operation, in a certain class of cases, but, owing to political wrangles, and to some extent to my inexperience, it died on the calendar.

I hope to make a second attempt to procure legislation on this subject, in the next Legislature, and my principal reason for wishing to bring the subject up at this time is to try to induce every member of this body to urge his local Senator and Representative to give this matter serious consideration.

[NOTE.—Dr. Parker, ex-county judge of Robertson county, is representing that county in the Legislature. The whole civilized world has awakened at last to a realization of the enormous evil of permitting the unlimited and indiscriminate production of idiots, imbeciles, lunatics and criminals to become an insupportable burden upon the taxpayers. The cost to the State of Texas in 1910 for the insane alone was, in round numbers, one million dollars, and every four years the State is compelled to provide additional buildings, equipment and officers to accommodate the ever-increasing horde of lunatics, hereditary alcoholics for the most part, and the number is increasing out of all proportion to population. Sterilization by the simple means proposed has become a pressing necessity, and already eight American States and one Canadian (Ontario)—realizing that such a measure is an extension of personal liberty and true humanity, as well as true economy—have enacted laws in accordance with the plan set forth in Dr. Parker's paper, towit: Indiana, California, Connecticut, Utah, Oregon, Iowa, New Jersey and New York, while the States of Louisiana, Florida and North Dakota will, this winter, consider such measure, upon petition of the State Associations of physicians. It is time Texas was falling into line and keeping up with the advance of civilization.—EDITOR TEXAS MEDICAL JOURNAL.]

